# An empirical study on social network analysis for small residential communities in Gangwon State, South Korea

**DOI:** 10.1038/s41598-024-62371-x

**Published:** 2024-05-22

**Authors:** Dae-hyun Jeong, Sang-Kyu Lee, Moo-Eob Ahn, Sang Mi Kim, Ohk-Hyun Ryu, Kyung Suk Park, Se Gye Shin, Jae Hyun Han

**Affiliations:** 1 Gangwon Institute, Chuncheon, Republic of Korea; 2 Hallym University College of Medicine, Chuncheon Sacred Heart Hospital, 77 Sakju-ro, Chuncheon, 24253 Republic of Korea; 3 Department of AI Health Information Management, Yonsei University, Wonju, Republic of Korea; 4 Industry Academic Cooperation Foundation, Hallym University, Chuncheon, Republic of Korea; 5 Gangwon Technopark, Chuncheon, Gangwon-do Republic of Korea

**Keywords:** Community network, Health promotion, Social network analysis, Health care, Medical research

## Abstract

Social Network Analysis (SNA) provides a dynamic framework for examining interactions and connections within networks, elucidating how these relationships impact behaviors and outcomes. This study targeted small residential communities in Gangwon State, South Korea, to explore network formation theories and derive strategies for enhancing health promotion services in rural communities. Conducted in 12 small residential areas, the survey led to a network categorization model distinguishing networks as formal, informal, or non-existent. Key findings demonstrated that demographic and socio-economic factors, specifically age, income, living environment, leisure activities, and education level, significantly influence network formation. Importantly, age, environmental conditions, satisfaction with public transportation, and walking frequency were closely associated with the evolution of formal networks. These results highlight the importance of early community network assessments, which must consider distinct network traits to develop effective health promotion models. Utilizing SNA early in the assessment process can improve understanding of network dynamics and optimize the effectiveness of health interventions.

## Introduction

Global trends show an increase in aging populations and urbanization^1^. In South Korea, particularly in rural areas, there is a notable presence of the elderly, aged 65 and over. These regions often exhibit lower socio-economic status, with reduced income and education levels, and a less healthy lifestyle characterized by lower rates of non-smoking, moderate drinking, and regular walking^2,3^. Rural communities face additional challenges such as sparse populations, vast geographical areas, inadequate public transportation, and limited health resources, deepening the health disparities with urban areas^4,5^. Furthermore, the Coronavirus Disease 2019 (COVID-19) pandemic has exacerbated social isolation and mental health issues like anxiety, depression, and stress due to reduced face-to-face interactions and weakened interpersonal bonds^6,7^.

Social Network Analysis (SNA) is a disciplined inquiry into the patterning of relations among social actors^8^. SNA enables the understanding of the structures and dynamics of relationships within social networks^9^. Additionally, SNA offers a robust framework for analyzing the interactions and connections within a network, revealing how these relationships influence behaviors and outcomes^10,11^. Effective network interventions highlight the strategic use of network insights from SNA to implement changes that foster stronger and more cohesive community ties^12^.

Collaborations with non-profit organizations and community networks are crucial for addressing diverse health and public service needs^13–15^. Such partnerships facilitate the sharing of resources and expertise, thus effectively meeting community demands, particularly in health promotion.

Previous studies on community networks have mainly focused on two aspects: the determinants of network formation and the dynamics of effective network structuring. Network formation is seen as crucial for addressing community needs, with ongoing research emphasizing the measurement of network centrality as a key factor in validating its importance. The primary aim of network formation is to enhance community problem-solving capabilities through collaborative efforts among members. Preliminary findings suggest that in larger networks, not all members are interconnected; typically, three to four subgroups are interlinked. While strong ties exist between these subgroups, intra-group connections tend to be weaker. This observation has led to the hypothesis that individuals with high centrality should build strong connections with leaders of other subgroups to ensure the efficient dissemination of critical information across the entire network, thereby enhancing service delivery throughout the community^10,12,14,16,17^.

The second research focus is on the sustainability of networks initially formed through personal ties. It has been proposed that these networks, while starting informally based on personal relationships, gradually become institutionalized over time. For such networks to remain sustainable, a balance between formal and informal structures is necessary^18,19^. However, empirical research on these dynamics is lacking in the South Korean regional context, which presents challenges in applying community-centered network theories and practices effectively.

To address these gaps, our study formulated and tested research questions derived from these two established perspectives. Firstly, we explored what factors influence community network formation in rural communities. Prior research suggests that community needs are met through network structuring; thus, we examined whether overall community satisfaction—including aspects like the living environment, public transportation, and leisure participation—affects network formation^13,14,18,20–22^. Secondly, we investigated the factors that contribute to the formalization of network structures. Following the insights of Goodman et al.^23^, who noted that younger networks based on personal relationships evolve into more institutionalized forms, we tested whether community satisfaction impacts this transition. Our findings aim to inform the development of a smart healthcare service that is tailored to the specific needs of the rural community.

## Methods

### Data collection

In this study, we implemented an empirical investigation of network formation in rural communities, focusing on 12 small residential communities in Gangwon State, South Korea. The research protocol was reviewed and approved by the Clinical Research Ethics Committee of Hallym University Chuncheon Sacred Heart Hospital (Approval Number: CHUNCHEON 2021-09-001-001) and was conducted in accordance with the Declaration of Helsinki (the Declaration’s principles ensure that research involving human subjects is conducted ethically, aligning with the values of respect for individuals, beneficence, and justice), which emphasizes ethical standards including informed consent and ethical treatment of research subjects.

The participants were adults aged 20 and above, residing in Gangwon State’s small rural residential communities. Eligibility criteria included an intent to use local public health facilities, willingness to engage with remote healthcare services, and the ability to comprehend and respond to the questionnaire effectively.

The survey ran from October to November 2021 and was completed by 206 participants. It covered various areas including health status self-assessment, medical history, socio-physical community environment, human network mapping, educational and economic activities, smoking and drinking habits, and assessments of physical and mental health. The questionnaire used in this study was based on the Korean Community Health Survey (KCHS) instrument, which is rigorously developed and widely used by government agencies to ensure comprehensive and reliable data collection. KCHS questionnaire is designed to collect detailed information on the health status, health behaviors, usage of health services, preventive health practices, environmental health, and healthcare utilization among the Korean population^24^.

**Figure 1:**
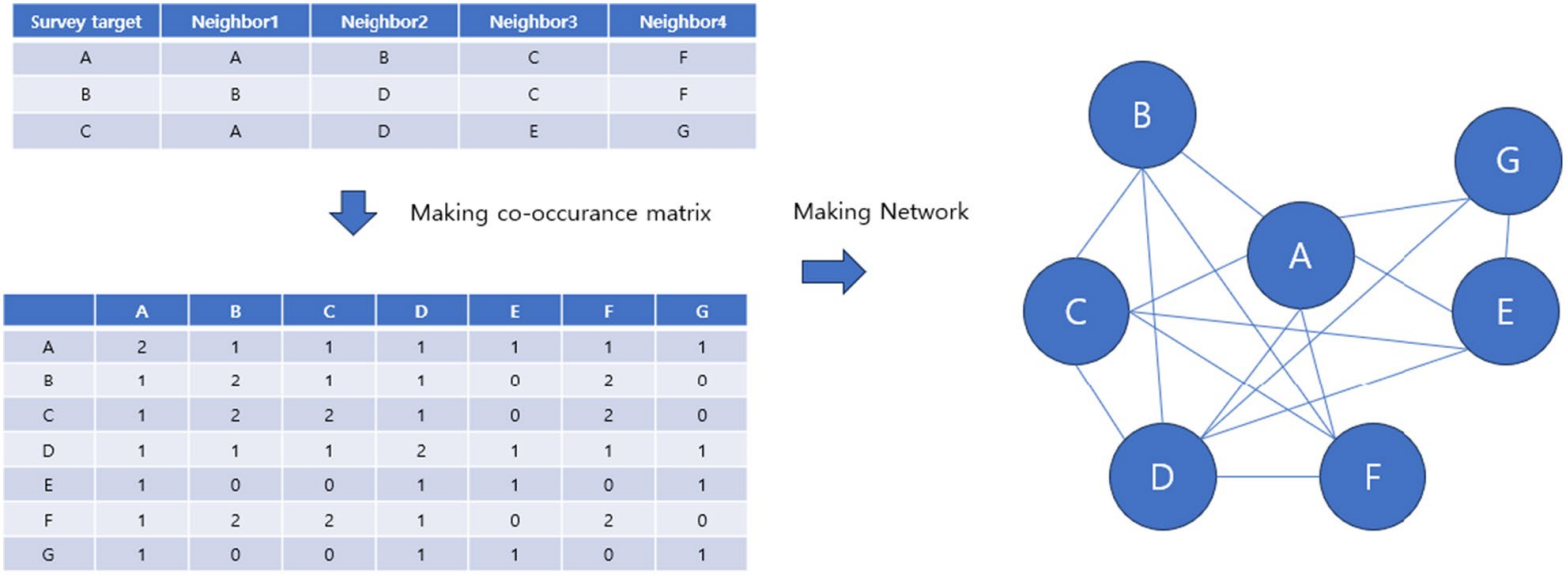
Example of a network system. Based on a survey where individuals marked their acquaintances among neighbors, a co-occurrence matrix was created, which ultimately led to the construction of the network.

To study human networks, we created and analyzed networks for individual communities based on initial survey data. We used a name generator method to identify members who recently interacted within each community, asking participants to list contacts from the last month to capture current and active connections^25,26^. We also had participants describe the nature of each interaction (familial, social, professional), helping to categorize and differentiate between formal and informal relationships. Our primary method was an egocentric network approach, using responses to map each participant's direct contacts and aggregating these to understand broader network dynamics. This method provides detailed insights into how individual interactions impact overall community structure. Based on this methodology, as illustrated in Fig. 1, we created a co-occurrence matrix for the members and used it to implement a network for each community. One of our primary network measurement methods was calculating degree centrality values. Degree centrality, an indicator identifying key hubs within the network, shows that members with higher centrality values are crucial for facilitating information exchange and opinion sharing within the community.

We constructed networks based on survey data to identify essential indicators and factors affecting network formation. For SNA, we utilized NetMiner 4.0 software (Cyram Inc., Seoul).

**Figure 2:**
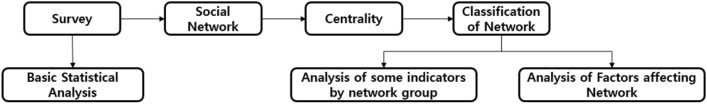
Research framework. Based on the survey, basic statistical analysis was conducted and, as shown in Fig. 1, network centrality was analyzed after the network implementation. Additionally, the forms of implemented networks (formal networks, non-formal networks, non-existing networks) were distinguished, and key indicators for each network, as well as major factors influencing the network, were analyzed.

In this study, social networks were categorized as formal, informal, or non-existent based on specific criteria developed from both the literature and empirical observations in the field (Fig. 2). Formal networks are characterized by structured relationships often involving organizational roles, official memberships, or documented interactions. Informal networks, on the other hand, consist of spontaneous, less structured interactions based on personal relationships without formal roles. Networks were considered non-existent in communities where no significant interactions were observed among members beyond casual acquaintance. Specifically, communities where ordinary residents exhibit high centrality were identified as informal networks, whereas communities where individuals hold formal roles, such as village heads, were classified as formal networks. Areas lacking measurable network centrality were defined as having no formed network. Table 1 summarizes the network patterns and characteristics of each community. This classification is grounded in social network theory as discussed in works by authors such as Granovetter on the strength of weak ties^18^ and Putnam^22^ on the role of social capital (Supplementary Fig. 1).

**Table 1:** Classification of network types for 12 small residential community in Gangwon state.

Classification	Standard	Group	Feature	Number of community
Network	Scale-free network	Formalized network	Members with high centrality have official titles	3
		Personal network	Members with high centrality are ordinary residents	4
Non network	Non scale-free network	Non-network	Centrality cannot be measured because it does not have a scale-free network form	5

**Figure 3:**
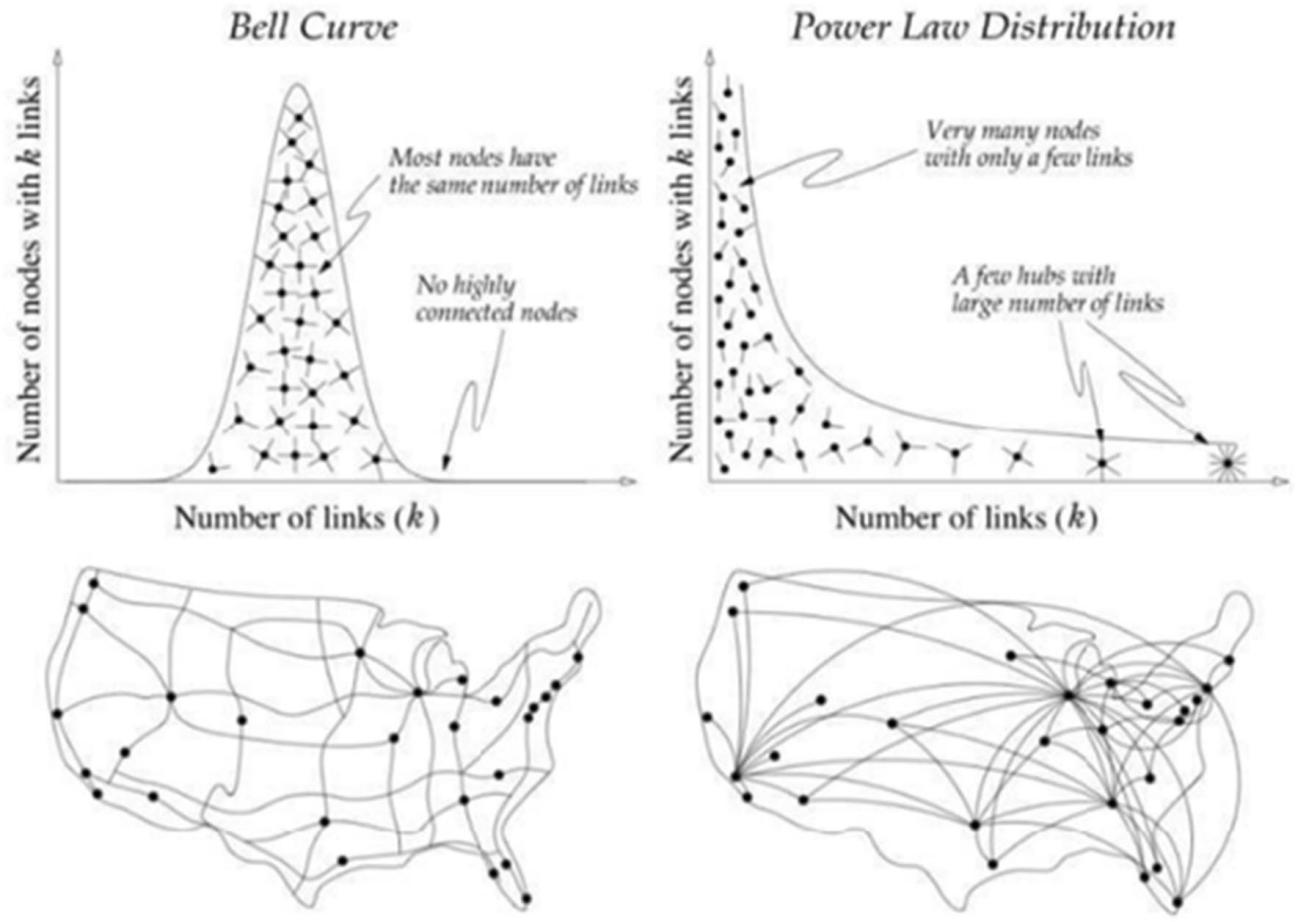
Normal and scale-free networks. Scale-free networks possess a power law distribution form, and this form of power law distribution features network hubs, similar to those seen in aviation systems.

Network analysis typically studies groups of actors, like individuals or organizations, across various domains, particularly in the social sciences. Multiple theories focus on network structures’ inherent characteristics. Identifying an area’s features based on its network type involves first establishing whether a ‘scale-free’ network exists. Introduced by Barabasi and Albert^27^, this model initially described internet web pages, suggesting that connections in random networks follow a power-law distribution, indicating hubs within the network, as shown in Fig. 3.

Scale-free networks are distinguished by their principles of growth and preferential attachment. The growth of such networks begins with a small number of nodes, expanding as new nodes are added. Preferential attachment refers to the tendency where new nodes are more likely to connect to nodes that are already well-connected. This phenomenon leads to the formation of network hubs, which are critical for determining the centrality within the network structure^27^.

### Statistical analysis

The data were analyzed using IBM SPSS Statistics for Windows (Version 27; Armonk, NY, USA). All statistical results were derived from a two-sided test, and a P-value less than 0.05 was deemed statistically significant. Continuous variables are presented as mean and standard deviation, while categorical variables are expressed in terms of frequency and percentage. Multiple logistic regression analysis was utilized to explore the factors influencing network formation and formalization within the small living area communities of Gangwon State, Korea.

## Results

Table 2 outlines the demographics of the 201 study participants, who had an average age of 62.9 years. Approximately half were aged 65 or older, 75% were female, and 40% met the Asia–Pacific criteria for obesity based on their body mass index. Furthermore, 75% of the participants were married. Our basic statistical analysis showed that communities with established networks typically had older residents, lower income levels, reduced satisfaction with the local living environment and public transportation, and generally lower education levels. Interestingly, these communities displayed a higher engagement in recent weight management efforts (see Table 3).

**Table 2:** Demographic characteristics of study participants.

Variables	Total (n = 201)
Age (years)	62.9 ± 11.7
< 50	24 (11.9)
50–64	80 (39.8)
≥ 65	97 (48.3)
Sex	
Male	52 (25.9)
Female	149 (74.1)
BMI (kg/m^2^)	24.3 ± 3.2
< 18.5	3 (1.5)
18.5–22.9	77 (38.7)
23.0–24.9	41 (20.6)
≥ 25	78 (39.2)
HTN	94 (46.8)
DM	32 (16.0)
Dyslipidemia	61 (30.3)
Education	
Unschooled	6(6.0)
Elementary school	45 (22.4)
Middle school	39 (19.4)
High school	61 (30.3)
College graduate or higher	44 (21.9)
Marital status	
Married	151 (75.5)
Widowed	27 (13.5)
Divorced	7 (3.5)
Separated	2 (1.0)
Unmarried	13 (6.5)

*BMI* body mass index, *DM* diabetes mellitus, *HTN* hypertension.

Values are presented as mean ± standard deviation for continuous variables and frequency (percentage) for categorical variables.

**Table 3:** Demographic, socioeconomic, and environmental characteristics of the group according to the presence or absence of a network.

Variables	Network formation		P-value
	Yes	No	
Age (years)	65.54 ± 10.51	60.76 ± 12.92	0.005
Average monthly income (above 3 million Korean won, %)	7.46	18.57	0.017
Satisfaction with living environment (yes, %))	61.94	82.61	0.002
Satisfaction with public transportation conditions (yes, %)	44.03	60.87	0.024
Leisure Participation (yes, %)	28.36	44.93	0.009
Education level (college graduate or higher, %)	18.38	28.57	0.005

*SD* standard deviation.

Values are presented as mean ± standard deviation for continuous variables and frequency (percentage) for categorical variables. P values were calculated t-test for continuous variables or Chi-square test for categorical variables.

In our logistic regression analysis, which aimed to identify factors influencing network formation, age, income, satisfaction with living and transportation conditions, leisure activity participation, and educational level were all found to be significant. Notably, dissatisfaction with community living conditions and public transportation significantly influenced the likelihood of network formation (Table 4).

**Table 4:** Factors influencing community network formation as identified by multiple logistic regression analysis.

Variables	Exp (B)	Cl (95%)	P-value
Age	1.037	1.010–1.064	0.006
Average monthly income	0.765	0.613–0.955	0.018
Satisfaction with the living environment	0.343	0.168–0.699	0.003
Satisfaction with public transportation conditions	0.532	0.295–0.957	0.035
Leisure participation	0.460	0.250–0.846	0.012
Education level	0.736	0.573–0.944	.016

*CI* confidence interval.

Further statistical analysis, segmented by group, revealed that the formal network group had the highest average age and the lowest levels of satisfaction with their living environment and public transportation. While their engagement in leisure activities was comparatively lower than other groups, their commitment to health-promoting activities, such as frequent walking, was more pronounced (Table 5).

**Table 5:** Characteristics of formalized, personal and non-network group.

Variables	Formalized network	Personal network	Non-network	P-value
Age	66.54 ± 7.95	64.97 ± 11.75	60.76 ± 12.91	0.014
Satisfaction with living environment (yes, %)	48.98	69.41	82.61	0.001
Satisfaction with public transportation conditions (yes, %)	31.25	51.76	60.00	0.008
Leisure Participation (yes, %)	28.00	28.57	46.27	0.041
walking days per week (weekly)	5.72 ± 1.91	4.54 ± 2.05	4.91 ± 0.94	0.006

*SD* standard deviation.

Values are presented as mean ± standard deviation for continuous variables and frequency (percentage) for categorical variables. P values were calculated t-test for continuous variables or Chi-square test for categorical variables.

Our logistic regression analysis to determine factors influencing the formalization of networks within these communities found age, satisfaction with living environment and public transportation, and average walking days per week to be influential. Particularly, marked dissatisfaction with the community's living environment and public transportation significantly impacted the formalization of networks (Table 6).

**Table 6:** Logistic regression model for factors affecting network formalization.

Variables	Exp (B)	Cl (95%)	P-value
Age	1.051	1.010–1.094	0.014
Satisfaction with living environment	0.423	0.205–0.874	0.020
Satisfaction with public transportation conditions	0.424	0.201–0.891	0.024
walking days per week (weekly)	1.051	1.010–1.094	0.014

## Discussion

In our study, we focused on 12 regions in Gangwon State to empirically investigate network formation within rural communities. We found that factors such as age, income, living environment, leisure activities, and education level influence network creation. Specifically, age, living environment, satisfaction with public transportation, and walking frequency were linked to the evolution of formal networks. Our findings indicate that areas with greater community needs, especially those less satisfied with basic community services, tend to form networks more actively. Notably, regions with limited capacities exhibited a stronger tendency to form networks, presumably to compensate for these deficiencies.

Our findings align with previous research, indicating that social networks play a critical role in health promotion and community resilience. As Fernández-Peña et al. highlighted in their systematic scoping review, SNA is invaluable for understanding social support dynamics within communities, particularly in how these networks provide care and facilitate health-related interventions^28^. This is particularly relevant to our study, as we explored the mechanisms through which network structure affects health services uptake in rural areas of Gangwon State.

Additionally, the systematic review and meta-analysis by Hunter et al. underscores the efficacy of leveraging network properties to enhance health behaviors and outcomes^29^. Our study contributes to this body of knowledge by demonstrating that network centrality and the formalization of networks can significantly impact community health practices, echoing Hunter et al.’s findings on the potential of structured network interventions.

Furthermore, Nickel and Knesebeck's research on community-based health interventions reveals the complexity of addressing health inequalities through networked community efforts^30^. Our research supports their conclusion that multi-faceted community interventions can effectively tackle health disparities, particularly when they leverage formal and informal networks to enhance service delivery and community engagement.

Lastly, Wolbring et al. provide a detailed analysis of community sports networks, which we found analogous to our study’s focus on health promotion networks. Their analysis of structural properties and cooperation conditions within networks offers valuable parallels to our findings, where the structural aspects of networks (e.g., centrality, tie formation) were crucial in mediating health outcomes^31^. By drawing on these parallels, we further substantiate our model of network-driven health promotion.

Previous research suggests that networks often formalize to ensure sustainability^20,21^. Our empirical data corroborates this, showing that areas with formal networks, as opposed to those formed through personal connections, generally reported lower satisfaction with critical community services. Additionally, these regions with formal networks often involved members who had limited individual capacities.

This study primarily involved participants aged 65 and above from rural community areas. Therefore, generalizing these findings to individuals under 65 or those residing in urban communities may be limited. Additionally, the selection of research areas and participants was not conducted through randomization. Instead, the study specifically targeted residents who showed an interest in non-face-to-face health management services and those who were connected to public health medical institutions within their communities. Therefore, it's important to acknowledge that the network analysis was predominantly conducted among residents with a heightened interest in health management and an inclination towards active participation in local public health initiatives. This targeted approach may influence the interpretation and applicability of the research results, as they may not reflect the perspectives or behaviors of a broader, more diverse population.

Our study revealed that networks in small living areas are formed to improve community living environment, especially in regions where members have limited individual capacities. We observed that influential community members, who play central roles in these networks, are vital for effectively disseminating healthcare information and services. Interestingly, areas with formal networks demonstrated better outcomes in recent health-promoting efforts compared to those without such networks. By integrating our findings with insights from related studies, we enhance the foundation of our network approach to improving health in rural communities. This discussion not only confirms the importance of our research but also highlights how using SNA can lead to innovative ways of delivering health services in rural area.

## Conclusion

In our study, we conducted a community survey across 12 small living areas in Gangwon State, utilizing social network analysis (SNA) to identify and characterize the networks present within each rural community. Our findings indicate that factors such as age, income, satisfaction with living conditions and public transportation, leisure activity participation, and education level significantly influence the formation of these networks. Furthermore, aspects like age, satisfaction with living conditions and public transportation, and the frequency of walking per week were closely linked to the formalization of these networks. However, the acute shortage of community health and medical resources in Gangwon State underscores the urgent need for efficient healthcare service provision. SNA emerges as a pivotal tool in this context, providing insights into community member relationships and interactions.This analytical approach is instrumental in developing and evaluating health promotion programs that foster active participation from residents and in understanding the mechanisms and impacts of various interventions.

Looking ahead, it is imperative for public healthcare services targeting small residential communities to incorporate preliminary assessments of community networks. This approach necessitates the development of healthcare service models that duly consider the unique characteristics of these community networks. Additionally, employing SNA in post-intervention evaluations can significantly aid in elucidating the mechanisms and effects of these health initiatives, thereby enhancing their effectiveness and relevance.

### Supplementary Information

Supplementary Figure 1.

## Data Availability

All data and materials will be provided upon individual requests to the corresponding author.
