# Supplementary material for: An empirical study on social network analysis for small residential communities in Gangwon State, South Korea
Source: Sci Rep. 2024 May 22;14:11648. doi: 10.1038/s41598-024-62371-x (PMC11109222; doi:10.1038/s41598-024-62371-x)
Supplement: Supplementary file 1 — Supplementary Figure 1. [file 41598_2024_62371_MOESM1_ESM.docx]

**Supplementary material**

|  | Network | classification |  | Network | Remark |
| --- | --- | --- | --- | --- | --- |
| 1 | 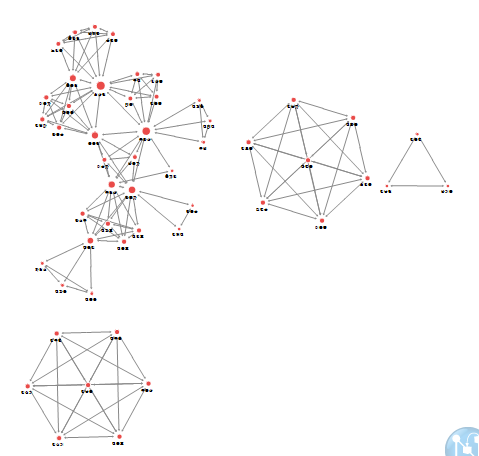 | Personal  network | 7 | 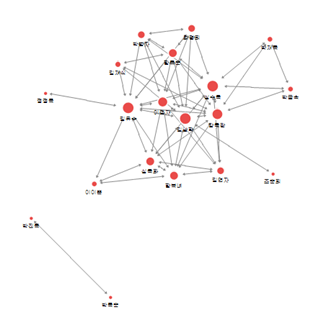 | Formalized network |
| 2 | 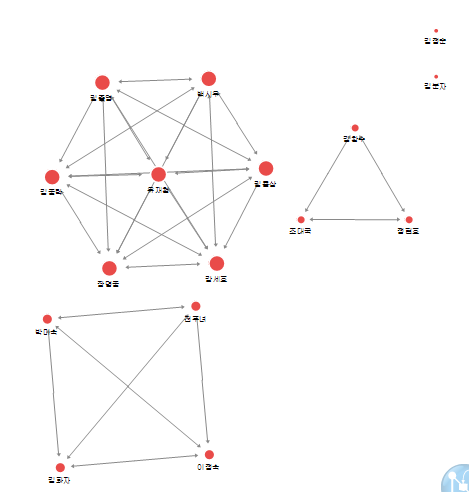 | Non-network | 8 | 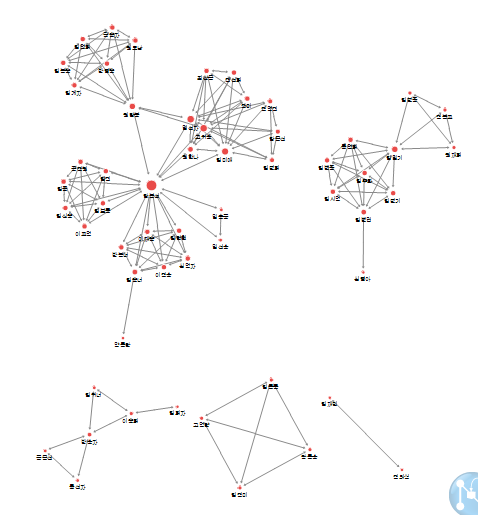 | Personal network |
| 3 | 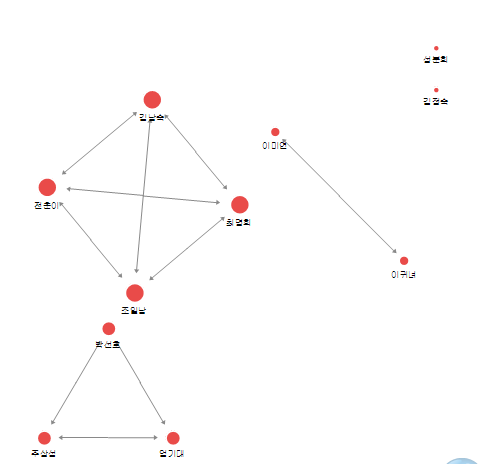 | Non-network | 9 | 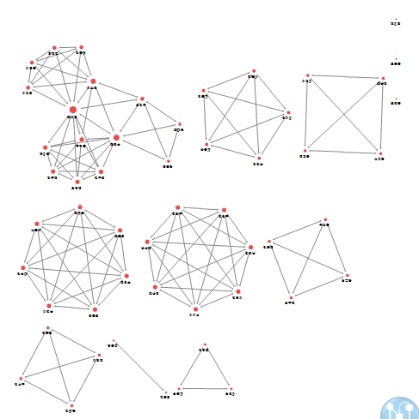 | Personal network |
| 4 | 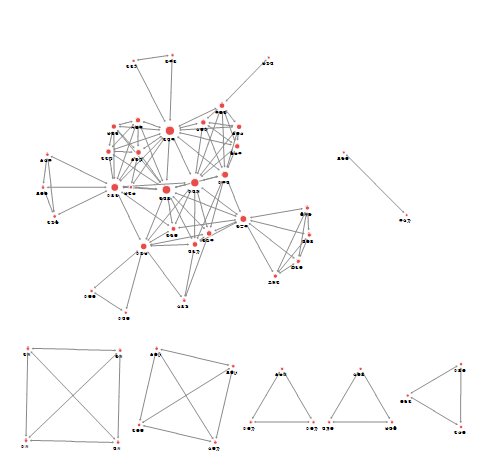 | Formalized network | 10 | 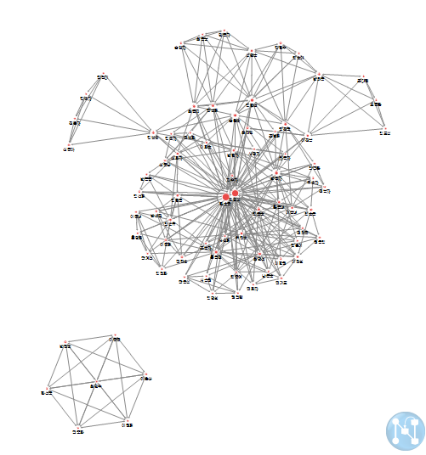 | Formalized network |
| 5 | 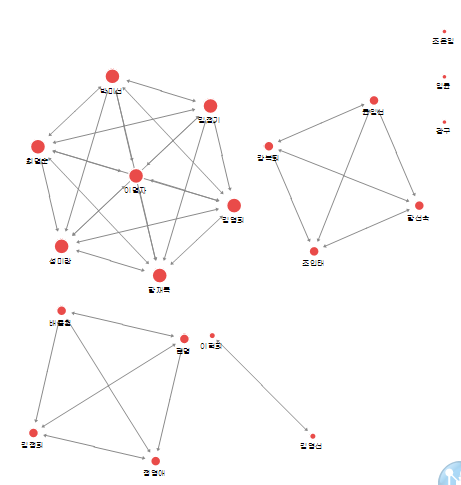 | Non-network | 11 | 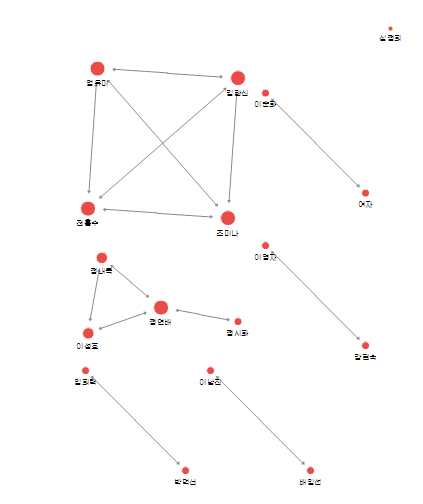 | Non-network |
| 6 | 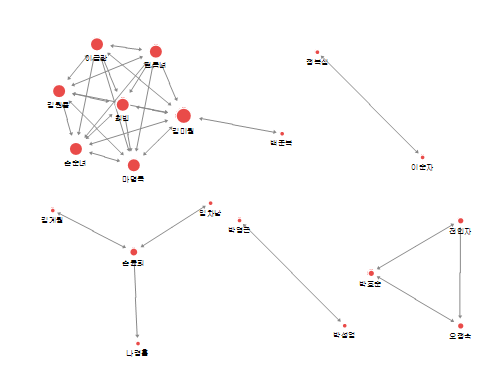 | Personal network | 12 | 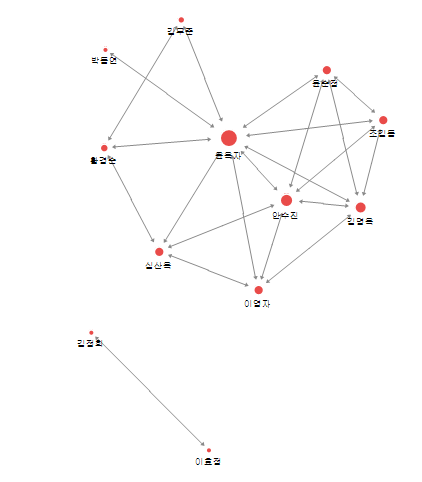 | Personal network |

Supplementary Fig.1. The networks of 12 communities
